# Faster Metabolite ^1^H Transverse Relaxation in the Elder Human Brain

**DOI:** 10.1371/journal.pone.0077572

**Published:** 2013-10-02

**Authors:** Małgorzata Marjańska, Uzay E. Emir, Dinesh K. Deelchand, Melissa Terpstra

**Affiliations:** Center for Magnetic Resonance Research and Department of Radiology, University of Minnesota, Minneapolis, Minnesota, United States of America; Northwestern University Feinberg School of Medicine, United States of America

## Abstract

^1^H magnetic resonance spectroscopy (MRS) is unique among imaging modalities because signals from several metabolites are measured during a single examination period. Each metabolite reflects a distinct intracellular process. Furthermore transverse (*T_2_*) relaxation times probe the viability of the cell microenvironment, e.g., the viscosity of the cellular fluids, the microscopic susceptibility distribution within the cells, and the iron content. In this study, *T_2_s* of brain metabolites were measured in the occipital lobe of eighteen young and fourteen elderly subjects at a field strength of 4 tesla. The *T_2_s* of *N*-acetylaspartate, total creatine, and total choline were 23%, 16% and 10% shorter in elderly than in young subjects. The findings of this study suggest that noninvasive detection of *T_2_* provides useful biological information on changes in the cellular microenvironment that take place during aging.

## Introduction

^1^H magnetic resonance spectroscopy (MRS) is unique among imaging modalities because signals from several metabolites are measured during a single examination period [1]. Each observable metabolite is associated with a distinct set of intracellular processes since the metabolites are primarily located in the intracellular compartment of the brain [1,2]. Some metabolites are preferentially concentrated in certain cell types and in different parts of the cell. *N*-acetylaspartate (NAA) is located primarily in neuronal cell bodies, axons, and dendrites, and is considered to be a sensitive marker for neuronal density or viability. Total creatine (tCr, creatine with phosphocreatine) and choline containing compounds (tCho, phosphorylcholine (PCho) and glycerophosphorylcholine (GPC)) are present both in neuronal and glial cells [1]. NAA and tCr are located in the cytoplasm [1,3,4], and tCho in the cytoplasm and cellular membrane [5]. Therefore, abnormal concentrations of specific metabolites may reflect particular aspects of neurodegenerative processes at molecular and cellular levels. However, detection of static metabolite concentrations alone provides only a partial description of viability.

Transverse relaxation results from nuclear spin-spin interactions and is sensitive to changes in molecular motion, mainly through interaction of metabolites with structural or cytosolic macromolecules [6,7]. Previous MRS studies of transverse relaxation time constants (*T*_2_s) of cerebral metabolites have observed changes induced by pathology or naturally occurring processes during development [6–9]. Hence, *T*_2_s of metabolites could be used to probe the cellular microenvironment e.g., the viscosity of the cellular fluids, the microscopic susceptibility distribution within cells, and the iron content. As such, investigation of the *T*_2_s of several metabolites has high potential for early stage sensitivity to processes that take place during aging. Another advantage of assessing the dynamic parameter, *T*_2_, is that it is not susceptible to signal normalization which can confound the MRS measurement of static concentration.

Most studies in humans have focused on measurement of the *T*_2_ of singlet resonances due to the relative experimental simplicity. In a handful of studies related to aging, *T*_2_ values have been measured for singlets of NAA, tCr, and tCho with contradicting results [10–13]. Among those, one study of the centrum semiovale that utilized a linewidth based approach and has not been validated or adopted since publication [12] found a longer *T*_2_ of NAA in elderly. A separate study of the occipital cortex that was complicated by overlap among water and metabolite signals [10] did not find any age associated differences in *T*_2_. The two remaining studies found no age dependence in the frontal lobe [13], and shorter *T*_2_s of NAA, tCr, tCho in the occipital cortex as well as other brain regions of elderly than adolescent subjects [11]. Most of these studies were performed at 1.5 T [10,12,13] while one was performed at 3 T [11]. All four studies were performed in a small number of subjects (at most 10 subjects per group) and with various techniques. While age associated *T*_2_ dependence could be brain region specific, the discrepancies among current findings warrant further study of this phenomenon.

The aim of this study was to measure metabolite *T*_2_s in a larger cohort of cognitively healthy young and elderly subjects than has been studied previously. Study of the occipital cortex facilitated examination of normal aging without confounding by pre-symptomatic presence of the most prevalent neurodegenerative conditions that impact other brain regions [14,15]. Data in this study were measured at a higher magnetic field, i.e., 4 T, which provides higher signal to noise ratio (SNR) and better spectral dispersion which leads to less overlap among resonances. Further SNR improvement was achieved using a dual loop receiver that could be used to study this superficial brain region. Additionally, each *T*_2_ was characterized using a larger number of echo times (*T*_E_s) than previously applied, and shorter *T*_E_s were sampled. Spectra were fitted and quantified using LCModel for robust characterization of the signal strength, and the macromolecule contributions were taken into account. Monte Carlo simulations were performed to assess the extent to which the measured *T*_2_ could be biased by the *T*_E_s utilized.

## Materials and Methods

### 1: Study population

Eighteen young (6 males, age 20 ± 1 (mean ± standard deviation (SD)) years, age range 18 to 22 years) and 14 elderly (7 males, age 77 ± 5 years, age range 70 to 80 years) individuals gave informed consent for this study, which was conducted according to the procedures approved by the Human Subjects’ Protection Committee at the University of Minnesota. Candidates with neurological disorders or chronic diseases were excluded. All recruited subjects provided written informed consent. The informed consent procedure included making clear to the participant that they did not have to be in this study in order to get treatment or benefits. We also made the voluntary nature of this study clear. It was not possible to deny any potential participant treatment or other advantages associated with this study because there were no treatments or benefits associated with this study (especially considering that the vitamin C was not given because it was associated with a different phase of the experimental design that was not completed per interim research findings and funding limitations). There was no direct or societal benefit to participation in this study. The Montreal Cognitive Assessment (MoCA) was administered to detect mild cognitive impairment with high sensitivity [16]. To detect more subtle changes, timed performances on the symbol digit modalities test (DSMT, Psychological Assessment Resources, Inc., FL, USA) and on the Trail Making Test (TMTA and TMTB) were also evaluated. Each subject completed the 30 question Geriatric Depression Scale (GDS) [17]. Average scores of young participants on cognitive tests were: 28 tasks completed successfully on the MoCA, 19 s on TMTA, 43 s on TMTB, 62 digits identified on the DSMT and 2 “yes” answers on the GDS. Average scores of elderly participants were: 27 on MoCA, 33 s for TMTA, 91 s for TMTB, 41 digits on the DSMT, and 3 “yes” answers on the GDS. As such, the young and elderly participants performed similarly on the MoCA and were not depressed, while the elderly subjects completed the timed tasks more slowly as expected.

### 2: MR acquisition

MR acquisitions were performed using a 4-T, 90-cm bore magnet (Oxford Magnet Technology, Oxford, UK) interfaced to Varian INOVA console (Varian, Palo Alto, CA). The magnet was equipped with a Sonata gradient coil (Siemens, Erlangen, Germany), which was capable of reaching 40 mT/m in 400 µs. A quadrature 169 MHz ^1^H surface radiofrequency (RF) coil was used to transmit and receive [18].

Each subject was positioned supine in the horizontal bore magnet with the RF coil subjacent to their occipital lobe. To ensure consistency in positioning, the external occipital protuberance, the bridge of the nose and the midline of the chin were used to define the median plane, which was centered in the bore such that midline of the brain was parallel to the axis of the magnet.

Localizer *T*_2_-weighted multislice rapid acquisition with relaxation enhancement (RARE) images [19] (repetition time (*T*_R_) = 4 s, *T*_E_ = 60 ms, echo train length = 8, matrix = 256x 128, slice thickness = 2 mm, 5 slices) were acquired to select a cubic volume of interest (VOI: 3 x 3 x 3 cm^3^) centered on the midsagittal plane in the occipital lobe. Linewidths of 12 ± 1.5 Hz for water were obtained in both groups with no significant difference between the groups after the adjustment of all first- and second-order shim currents using the fast automatic shimming technique by mapping along projections with echo planar imaging readout (FAST(EST)MAP) [20,21].

Spectra were acquired using the stimulated echo acquisition mode (STEAM) sequence [22,23] with water suppression via variable-power RF pulses with optimized relaxation delays (VAPOR) and outer volume saturation [24]. Spectra were collected at the following seven *T*_E_s: 10, 20, 30, 40, 60, 80, and 180 ms. Four averages were obtained for each *T*_E_ with a repetition time (*T*_R_) of 4.5 s and STEAM mixing time of 42 ms. Additionally, unsuppressed water spectra were acquired from each subject at the following *T*_E_s: 5, 10, 20, 30, 50, 100, 200, 400, 800, 1500, 3000, 5000 ms with *T*_R_ of 15 s. Spectra were acquired with 6,144 complex points and a spectral width of 6 kHz. Macromolecule (MM) spectra were measured from two subjects using the inversion-recovery (metabolite-nulled) technique [25] at all *T*_E_s except 180 ms, at which *T*_E_ the MM signal was too weak for observation (*T*_R_ = 2 s, inversion time (*T*_I_) = 0.69 s, 64 averages for *T*_E_s 10, 20, 30, and 40 ms, 128 averages for *T*_E_s 60, and 80 ms].

### 3: Spectral processing and fitting

MR spectra were analyzed using LCModel version 6.1-4A [26,27] (Stephen Provencher, Inc., Oakville, Ontario, Canada). The basis set for LCModel was generated using home-written Matlab (The MathWorks, Inc., Natick, MA, USA) simulations based on the density matrix formalism [28] and known chemical shifts and *J*-coupling constants [29,30]. The simulated spectra of the following twenty one metabolites were included in the basis set: alanine (Ala), ascorbate (Asc), aspartate (Asp), creatine (Cr), γ-aminobutyric acid (GABA), glucose (Glc), glutamine (Gln), glutamate (Glu), glycerophosphorylcholine (GPC), glycine (Gly), glutathione (GSH), lactate (Lac), *myo*-inositol (*m*Ins), N-acetylaspartate (NAA), *N*-acetylaspartylglutamate (NAAG), phosphocreatine (PCr), phosphorylcholine (PCho), phosphorylethanolamine (PE), scyllo-inositol (*s*Ins), taurine (Tau) and threonine (Thr). Five metabolites, NAA, Cr, PCr, PCho, and GPC were separated into constituent singlet and multiplet moieties. Flags were set for calculation of tCr, i.e. combined singlet moieties of Cr and PCr, and tCho, i.e. combined singlet resonances of PCho and GPC. The occipital cortex MM spectra (average from two subjects) measured at all *T*_E_ except 180 ms (i.e. whence the MM signals were negligible) were included in the basis set.

No baseline correction, zero-filling or line broadening were applied to the *in vivo* data before input into LCModel. The fitting parameter that controls the flatness of the spline baseline function (DKNTMN) was set to 0.25, and spectra were fitted over the 0.5 to 4.2 ppm range.

### 4: *T*_2_ and CSF analysis

The *T*_2_ values were determined by fitting the metabolite concentrations obtained from LCModel analysis (without water scaling) as a function of *T*_E_ using a two parameter, mono-exponential decay function with a nonlinear least square algorithm in Matlab. The *T*_2_ fitting was only performed on metabolite concentrations that were estimated reliably (i.e. the Cramer-Rao lower bound (CRLB) estimate of percent standard deviation of the concentration was less than 30%) at all seven *T*_E_s. The integrals of unsuppressed water spectra were fitted with biexponential decay function with free parameters being *T*_2_ of tissue water and cerebrospinal fluid (CSF) content of the VOI [31].

### 5: Monte Carlo simulations

Monte Carlo simulations were used to test for bias in calculating *T*_2_s from spectra that were measured in this study. As such, for each measured *T*_2_, signal strengths were computed theoretically at all *T*_E_s used in *in vivo* experiments as well as at the additional *T*_E_s that were not studied *in vivo* (120, 250, 400 ms). To account for the actual SNR of the experimental *in vivo* spectra, the random noise utilized in each Monte Carlo simulation was based on the maximum residual between measured *in vivo* data points and fitted *T*_2_ curves. For each of 500 iterations per T_2_, random noise (constrained to the maximum % *T*_2_ fit residual measured per metabolite and age group from the experimental data) was added to the theoretically computed signal intensity at each *T*_E_, and *T*_2_ was fitted to the noised points with the two-parameter, single-exponential function. The associated SD was also determined based on the goodness of the nonlinear fit.

### 6: Statistical analysis

Statistical analysis was conducted using SAS Software for Windows (version 9.1, SAS Institute, Cary, NC, USA). Normal distribution could not be assumed due to heteroscedasticity, therefore a nonparametric test was chosen for analysis. The *T*_2_ relaxation times of each metabolite and tissue water were compared between young and elderly cohorts using a Wilcoxon two sample test. The findings were considered significant if *p* < 0.05. No attempt was made to correlate *T*_2_s with age or cognitive status due to the narrow range in age and cognitive scores.

## Results

**Figure 1:**
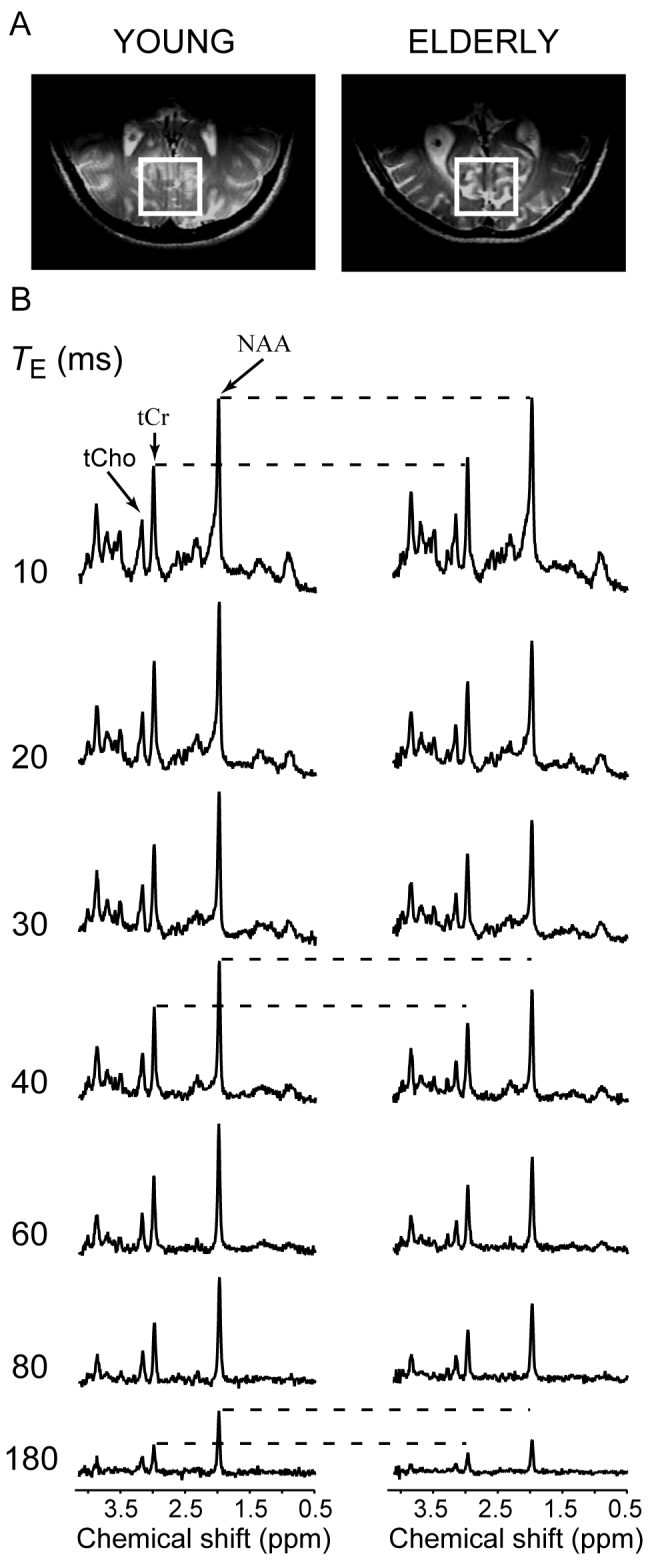
Voxel placement and data quality. (A) Images of human brains illustrating the position and size of the VOI. (B) Representative ^1^H STEAM spectra (4 T, 27 mL, *T*_R_ = 4.5 s, number of averages = 4) measured at seven *T*_E_s from the human occipital lobe in one young (left) and one elderly (right) subject. The vertical scale has been adjusted such that the NAA resonance detected at *T*_E_ = 10 ms for both young and elderly subjects has the same intensity. Horizontal dashed lines are visual guides to indicate that the intensity of NAA and tCr signals decrease faster in the elderly than the young subject. The faster signal decay reflects a shorter *T*_2_ value. Spectra are shown without line broadening. NAA, N-acetylaspartate, tCr, total creatine = creatine (Cr) + phosphocreatine (PCr), tCho, total choline = choline containing compounds.

Representative ^1^H spectra obtained at all seven *T*_E_s from one young and one elderly subject are shown along with the position of the VOI in Figure 1. The CSF content of the VOI was significantly different between elderly and young subjects (25 ± 11% vs. 9 ± 3%, *p* < 0.0001). The excellent spectral quality that was achieved in all subjects was characterized by high SNR, narrow linewidths, absence of contamination form signals outside the voxel, and effective water suppression. Changes in resonance intensities and patterns can be observed as a function of *T*_E_ for both young and elderly subjects. The singlet resonances such as NAA at 2.01 ppm, tCr at 3.03 ppm, and tCho at 3.2 ppm become smaller with increasing *T*_E_ whereas the multiplet resonances not only become smaller but also undergo *J*-modulation. The decay of singlet signal intensity with increasing *T*_E_ is more pronounced in the elderly subject than the young subject. For better visualization of this phenomenon, the vertical scale has been adjusted such that singlet NAA resonance measured at *T*_E_ = 10 ms in both the young and the elderly subject has the same intensity. The horizontal dashed lines clarify that the NAA and tCr intensities are lower for elderly subject than the young subject at *T*_E_s of 40 and 180 ms. The faster signal decay reflects a shorter *T*_2_.

**Figure 2:**
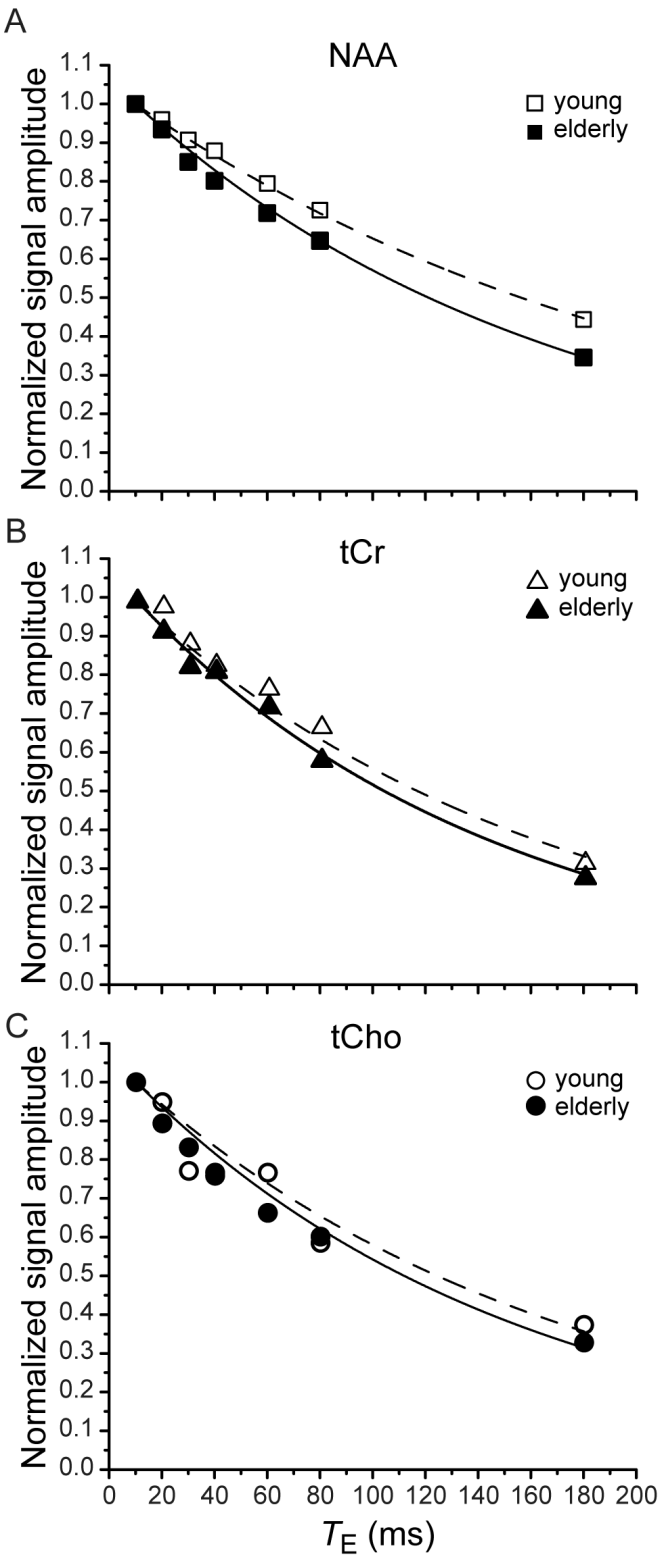
*T*_2_ fits for metabolites in young and elderly subjects. Individual exponential fits (represented by decaying lines) of the experimentally measured data for (A) the NAA singlet at 2.01 ppm, (B) the tCr signal at 3.03 ppm, and (C) the tCho singlet at 3.2 ppm in one representative young and one representative elderly subject. The amplitude of all data sets was normalized by setting the first *T*_E_ point to unity for both young and elderly subjects. For all metabolites and all subjects, *T*_2_s were fit with R^2^ ≥ 0.918, with the lowest R^2^ for tCho.

Representative *T*_2_ exponential fits for the singlet NAA, tCr and tCho resonances in one young and one elderly subject are shown in Figure 2. These are the only metabolites in the basis set that satisfied quality control criteria. All fits were of good quality with R^2^ values (indicators of the accuracy of the exponential fits) ≥ 0.918. The elderly curves fall below the young curves for all three metabolites illustrating the consistently faster relaxation in the elderly subject. The extent to which signal intensity drops off more quickly and to a larger extent is most apparent for NAA, demonstrating the greatest difference between elderly and young *T*_2_s for NAA among the three metabolites. The fit of the tCho data to exponential decay was not as good as for other compounds, leading to a lower precision in measuring *T*_2_.

Table 1 summarizes *T*_2_ values of NAA, tCr, tCho, and tissue water for all subjects. The *T*_2_s were significantly shorter using a one-sided probability distribution in elderly than the young subjects by 23%, 16% and 10% for NAA, tCr, tCho, respectively. When a two-sided probability distribution was used, the difference in the *T*_2_ of tCho between young and elderly subjects did not reach significance. Additionally, *T*_2_ of tissue water was significantly shorter for elderly subjects as compared to young subjects.

**Table 1:** *T*_2_ in young and elderly cohorts and summary statistics.

Compound	Young *T*_2_ (ms)	Elderly *T*_2_ (ms)	One-sided probability *p*	Two-sided probability *p*	Elderly *T*_2_ ∕ Young *T*_2_	
NAA	208 ± 18 (252 – 176)	161 ± 19 (200 – 138)	0.00000014	0.00000028	0.77	
tCr	157 ± 14 (190 – 138)	132 ± 11 (154 – 120)	0.0000044	0.0000087	0.84	
tCho	164 ± 29 (248 – 131)	147 ± 16 (178 – 122)	0.0297	0.059	0.90	
Tissue water	52 ± 2 (48 - 57)	47 ± 4 (40 - 51)	0.0000019	0.0000039	0.90	

*T*_2_ values (mean ± SD, minimum and maximum values in parenthesis) NAA at 2.01 ppm, tCr at 3.03 ppm, tCho at 3.20 ppm, and tissue water measured in the occipital lobe in 18 young and 14 elderly subjects.

For all metabolites and both age groups, the *T*_2_ outcomes from the Monte Carlo simulations preformed with the *T*_E_s utilized experimentally were well within one standard deviation of the *T*_2_ generated utilizing three additional *T*_E_s. The standard deviations of the *T*_2_s simulated using the seven *T*_E_s utilized to measure the *in vivo* data were the same as those achieved experimentally. Lower standard deviations among simulated *T*_2_s were found when any single *T*_E_ was added to the data set. The extent of this improvement was similar for addition of either the 120 ms or the 250 ms *T*_E_. The greatest improvement was obtained by including all three extra *T*_E_s (120, 250, and 400 ms).

## Discussion

The current results indicate that aging has a significant effect on the *T*_2_ of metabolites, which suggests that this variable may be sensitive to intracellular changes that take place during the neurodegenerative process. Furthermore, this parameter should be considered when studying MRS measured metabolite concentrations among cohorts of differing ages. Although atrophy of the whole brain takes place during aging and CSF content of the VOI was significantly higher for elderly as compared to young subjects, the CSF content of VOI would not influence the measurement of *T*_2_ of metabolites due to the negligible concentration of metabolites in the CSF [32]. The *T*_2_s of all detected intracellular metabolites, NAA, tCr, tCho, and tissue water were shorter in the elderly cohort. The *T*_2_ values of NAA and tCr were significantly different between young and elderly subjects independent of the hypothesis (*p* < 0.05 for both two-sided and one-sided probability distributions). The largest difference was obtained for NAA with 26% shorter *T*_2_ in elderly as compared to young subjects. For tCr, the difference was 16%. Although a 10% shorter *T*_2_ was observed for tCho in elderly as compared to young subjects, this difference was only at the trend level when using a two-sided probability distribution (*p* = 0.059). This is due to large standard deviation among tCho *T*_2_s especially in young cohort. However, the difference was statistically significant under the hypothesis that lower *T*_2_ values will be observed in elderly than young subjects for all metabolites (one-sided probability distribution, *p* = 0.03). The standard deviation is higher for young subjects than elderly subjects due to the poorer fits.

The *T*_2_ measurements were performed in 2.1 minutes which is short enough to be routinely added to any spectroscopic protocol. However, with 4 scans taken at each *T*_E_, it was not possible to obtain *T*_2_ values for metabolites other than NAA, tCr and tCho due to the fact that reliable quantification of other metabolites was not possible at all *T*_E_s. The reliability in quantification of metabolite concentrations increases with increasing SNR [23]. Therefore, additional metabolites such as *m*Ins, Glu and Gln could potentially be quantified with an increased number of scans. Detection of the *T*_2_s of these additional compounds would increase the sensitivity of this approach to changes that take place during the neurodegenerative process.

The Monte Carlo simulations revealed that the same *T*_2_ values as obtained in this study would be measured if more *T*_E_s were added. Therefore, the *T*_2_ values observed for young and elderly subjects were not biased by the *T*_E_s at which spectra were measured. Additionally, Monte Carlo simulations revealed that the standard deviation values that were obtained experimentally were the lowest that could be expected based on the quality of the data used. Acquiring additional spectra at either one or two longer *T*_E_s than those used in this study would lower standard deviations, but not change the group mean *T*_2_. Acquiring this additional data would improve the accuracy of the *T*_2_ measured in each individual. Expected improvement in standard deviations would make findings more significant which would be especially appealing in the case of tCho. As such, the approach would become sensitive to changes in cell viability at an earlier disease stage. Nonetheless, the improvement would come at the cost of extending the measurement time since additional spectra would need to be acquired.

The *T*_2_ values measured in the young subjects were similar to those previously reported at the similar field strength [33–35]. Although the measurements were neither performed with the same methods nor in the same brain region, *T*_2_ values from all these studies are within one standard deviation of each other.

The *T*_2_ values of brain metabolites as a function of age have been measured in several studies at 1.5 or 3 T with contradicting results [10–13]. Our finding of faster *T*_2_s in elderly than young subjects is in agreement with the prior study that is most similar to ours [11]. Both of these studies scanned the occipital lobe, were carried out at similar magnetic field (3 and 4 T), and spanned approximately 60 years of aging (although the study by Christiansen et al. also spanned similar age range). At lower field, the smaller chemical shift dispersion leads to increased overlap among metabolites, which precludes separation of constituent signal contributions. For example, the NAA signal at 2.01 ppm has contributions from underlying resonances from Glu, Gln, and macromolecules. In the study by Kirov et al., the extent to which *T*_2_ was lower in elderly than adolescent subjects was: 12% for NAA, 10% for tCho, and 6% for tCr. These *T*_2_ values were obtained with a *T*_E_ set that was tailored to measuring the ~180 ms *T*_2_. In our study, shorter *T*_E_s were sampled which could be advantageous to extent to which differential information exists in the data at shorter *T*_E_s. Both studies observed the largest difference in *T*_2_ of NAA. They also agree that if a linear decline is assumed, the changes in *T*_2_s are less than 1 ms per year. The other study that was similar to ours in that spectral deconvolution for determination of constituent signal strengths was utilized did not find an age associated difference in the frontal cortex over a shorter age span [13]. The earliest study of this type [10] suffered from an unfortunate combination of utilizing the area under the peak to measure signal strength and persistence of substantial water signal in the metabolite spectra. While utilizing the integral area under the metabolite resonance generally suffers from confounding by overlapping resonances, the persistent substantial water signal may have exacerbated this problem. Finally, our study did not agree with novel study that was designed to measure metabolite concentrations with correction for relaxation rates [12]. Regarding that novel study, the authors cautions that “apparent age-dependence of metabolite *T*_2_s could merely be a reflection of age-dependent changes of signal characteristics of myelin water [12].” They also emphasized that the approach was designed to move toward true absolute quantification by taking into account non-negligible differences in relaxation rates. The approach used for measuring *T*_2_ in that work [12] has not been adopted since its publication. While the importance of considering possible age and disease associated difference in *T*_2_ continues to be emphasized, the need for dedication of additional scanning time to measure spectra at several *T*_E_s is generally acknowledged. Additional studies designed to scan the brain regions and age spans represented in these five studies as well as magnetic field dependence would be needed to further understand the origin of the discrepancies.

The human brain undergoes morphologic and physiological changes over the 50 to 60 years separating the two groups observed in this study. Since atrophy in normal aging involves neuronal shrinkage, as well as axonal and myelin degeneration [36–38], the resulting increase in the fraction of small neurons and reduction in water content could result in shorter *T*_2_ of metabolites [11]. Furthermore, age-dependent iron deposition has shown a strong correlation with *T*_2_ shortening [39,40]. The measurement of *T*_2_ of metabolites is not able to distinguish among these possible causes of *T*_2_ shortening. However, all of the phenomena take place in aging and at a younger age in Alzheimer’s disease. The cellular changes and iron accumulation occur regionally with some brain regions highly vulnerable to aging while other brain regions are spared. Therefore the brain region selectivity of this approach renders it highly applicable to for changes that occur in areas that are impacted earliest and to the greatest extent in Alzheimer’s diseases. That *T*_2_ differences between young and elderly subjects were found even though the occipital cortex is spared suggests that *T*_2_ measurement is sensitive to small changes in the cellular environment. *T*_2_ differences are expected to be largest in the brain regions that are most vulnerable to neurodegenerative processes.

## Conclusions

The findings of this study suggest that noninvasive detection of *T*_2_ provides useful biological information on changes in the cellular microenvironment that take place during aging. As such, brain region specific studies of *T*_2_ relaxation could lead toward discovery of an early stage biomarker, and could enlighten changes that take place during normal and pathological aging. Such studies could be designed to elucidate mechanisms, especially if combined with diffusion weighted spectroscopy and mapping of the regional iron content. *T*_2_ also has potential for development as a tool to monitor efficacy of experimental therapies.
