# Correction: Faster Metabolite ^1^H Transverse Relaxation in the Elder Human Brain

**DOI:** 10.1371/journal.pone.0092755

**Published:** 2014-03-07

**Authors:** The PLOS ONE Staff

There is an error in the last sentence of the first paragraph of the sub-heading “Spectral processing and fitting” under the “Materials and Methods” section. The correct sentence for this section is:

“The occipital cortex MM spectra (average from two subjects) measured at all TE except _60, 80 and_ 180 ms (i.e. whence the MM signals were negligible) were included in the basis set.”
